# Outnumbered: Control Prothrombin Time in Maddrey’s Discriminant Function Impacts Steroid Use but Not Mortality in Alcoholic Hepatitis

**DOI:** 10.3390/biology11121833

**Published:** 2022-12-16

**Authors:** Marcus Healey, Richard K. Sterling

**Affiliations:** 1 Internal Medicine, Virginia Commonwealth University (VCU) Health System, Richmond, VA 23219, USA; 2 Division of Gastroenterology, Hepatology, and Nutrition, Virginia Commonwealth University (VCU) Health System, Richmond, VA 23219, USA

**Keywords:** alcoholic hepatitis (AH), prothrombin time (PT), Maddrey’s discriminant function (MDF), steroids, model for end-stage liver disease (MELD)

## Abstract

**Simple Summary:**

Alcoholic hepatitis is associated with high morbidity and mortality. Maddrey’s discriminant function has been classically used to define the severity, with scores that are greater than 32 indicating poor outcomes and consideration for treatment with steroids. In order to use this calculation, a control prothrombin time is needed, which is not standardized across different electronic medical records or institutions. In this study, we aimed to determine if the control prothrombin time that is chosen has any effect on steroid use and mortality. We found that the higher the value of the control prothrombin time that was used, the less likely it was that individuals with alcoholic hepatitis would receive steroids. However, we found that this did not affect mortality in those who received steroids. This research is novel and important for providers to know when using Maddrey’s discriminant function, as the control PT values that they choose do have implications for treatment. Our findings also suggest that the model for end-stage liver disease (MELD) seems to be a better indicator of 30-day survival in this population.

**Abstract:**

Background and aims: In alcoholic hepatitis (AH), increases in the total bilirubin (TB) and the prothrombin time (PT), which are included in the Maddrey’s discriminant function (MDF) and the model for end-stage liver disease (MELD), are associated with poor outcomes. However, the impact of which control PT in the MDF to use compared to the MELD on the outcomes in AH is unknown. Our aim is to determine whether the choice of the control PT used in the MDF calculations has any impact on steroid use and survival when compared to the MELD in those with AH. Methods: Through retrospective chart review, we analyzed 882 subjects who were admitted from 2012 to 2020 with acute AH. Their MDF was calculated [(TB + 4.6 × (PT–control)] using the following three different control PTs: 12, 13.5, and 14.8 s, and was compared to the MELD. The primary outcomes were steroid use and 30-day survival. Results: When it was stratified by the control PT, the percentage of MDF ≥ 32 (the threshold for steroids) decreased with increasing control PT (70%, 61%, and 52%, respectively), along with decreased steroid use (91%, 84%, and 75%, respectively). Those who received steroids were not shown to have improved 30-day survival compared to those who did not receive steroids (*p* = 0.41). The ability of the MDF for each control PT threshold to predict 30-day survival was similar (AUROC 0.735), and was lower compared to the MELD (0.767). Conclusion: While the choice of PT control in the MDF impacted the use of steroids in AH, the use of steroids and the choice of PT control used did not impact the overall survival. Regardless of which control PT was used in the MDF, the MELD was better at predicting 30-day survival. Important information: Background: Treatment with steroids is indicated in alcoholic hepatitis (AH) with Maddrey discriminant function (MDF) ≥ 32 and the model for end-stage liver disease (MELD) ≥ 20. The impact that the control prothrombin time (PT) value that is used in MDF has on steroid use and survival in AH is poorly understood. Findings: The choice of control PT that is used when calculating the MDF impacts the use of steroids but does not impact mortality. The MELD was better than the MDF at any control PT used in predicting survival in acute AH. Implications for patient care: Providers should be aware that higher control PT’s have an effect on treatment decisions but should not generally impact survival in this population. The MELD appears to better predict 30-day survival in this population.

## 1. Introduction

Alcoholic hepatitis (AH) is a clinical syndrome with both clinical and laboratory derangements that are associated with a 30–40% mortality rate at one month in those with severe disease [1]. While excessive alcohol use within the last 60 days is a requirement for diagnosis, other criteria, such as serum total bilirubin (TB) > 3 mg/dL and an aspartate aminotransferase (AST) to alanine aminotransferase (ALT) ratio > 1.5 have also been established in order to determine which patients have AH [2]. While many methods exist to define AH, it is crucial to stratify the disease in terms of severity as it has implications for both treatment and mortality.

Several scores exist to risk stratify AH patients, including Maddrey’s discriminant function (MDF), the model for end-stage liver disease (MELD), the Glasgow alcoholic hepatitis score, and the age-bilirubin-international normalized ratio-creatinine score. Among these scores, the MDF and the MELD are the most used in the United States. The newest guidelines recommend the use of steroids (prednisolone 40 mg per day) orally for severe AH in the absence of contraindications for MELD scores > 20 and MDF scores > 32 [3].

Maddrey et al. created MDF in order to have a standard and easy-to-use method to predict the use of steroids and 28-day mortality in 1978 [4]. The prothrombin time (PT) was identified as an independent predictor of poor prognosis and, thus, would benefit from oral prednisolone therapy. However, in a subsequent multicenter study, because different laboratories had different thresholds regarding PT, a modified MDF was developed [(TB + 4.6 × (PT–control)] that includes the patient’s PT in reference to a standard control PT for that specific center [5]. However, the control PT for each of the centers in this study was not provided.

Studies to date have looked at different control PTs across institutions in order to determine the need for steroid therapy in AH [5]. However, to our knowledge there have been no studies that consider whether the choice of which control PT to use impacts the use of steroids and, therefore, the survival of the patients with AH. Moreover, recent data suggest that the MELD is superior to the MDF in predicting steroid use and mortality in AH [6]. Our aim in this study is to determine whether the specific value of the control PT that is used to calculate the MDF has an effect on whether steroids are used and the overall mortality in AH. We also compared the MDF (based on three control PT values) to the MELD on 30-day survival with or without steroid use.

## 2. Methods

A retrospective chart review was performed on an electronic medical record (EMR) from a large university institution for patients given the ICD-9 (571.1) and ICD-10 (K70.1) code criteria of AH from 1 January 2012 to 30 December 2020 under institutional review board (IRB) approval, with a waiver for consent. From this review, 2688 patients had a diagnosis of AH. Of these total encounters, 1806 were excluded, leaving 882 subjects with available data on index admission. The criteria for exclusion were not being admitted for AH, an emergency department visit without admission for AH, or missing PT or TB values.

The criteria to diagnose AH were based on the clinical judgement of the admitting team, who used the National Institute for Alcohol Abuse and Alcoholism (NIAAA) definitions of AH to guide their decision to use steroids or not. The NIAAA definition of probable AH includes a recent onset of jaundice within 8 weeks of admission, persistent alcohol consumption greater than 40 g and 60 g daily for females and males, respectively, for more than 6 months of time, which had to be within the last 60 days before admission. Important laboratory parameters include AST > 50 U/L and AST/ALT > 1.5 [2]. The exclusion criteria included uncontrolled infection, which included sepsis, septic shock, or other signs of infection, including positive blood cultures carried out on admission. If active gastrointestinal bleeding was present, an endoscopy was performed, and the patient was reassessed for steroids at day 7. Anemia and hemoccult-positive stool tests were not absolute contraindications to the diagnosis of AH or steroid use. Multiorgan failure and an alternative explanation for acute liver injury or liver failure were also exclusion criteria.

All 882 patients had their MDF calculated [(TB + 4.6 × (PT–control)] using three different control PT values of 12 s (low end of normal), 13.5 s (middle of range), and 14.8 s (high end of normal). The general demographic data was obtained to include parameters such as age, race, sex, ethnicity, and weight. The liver fibrosis (FIB-4) index and the labs pertaining to MDF and MELD calculations were collected. The clinical disease states on admission were reviewed and included history of hepatic encephalopathy (HE), diabetes mellitus, hepatitis B virus (HBV), hepatitis C virus (HCV), ascites, gastrointestinal bleed, and bacteremia. Other important clinical factors in this study were the percentage who died from AH on index admission and the total hospitalization in days for AH. The demographic data, clinical disease states, and other clinical factors mentioned above were analyzed in the context of those who received steroids and those who survived at least 30 days compared to no steroids and death, respectively.

All continuous data were assessed for normality and presented as mean (standard deviation) or median (interquartile range) as appropriate and compared using parametric (*t*-test) or nonparametric (Wilcoxon test). The categorical data were presented as proportion and were analyzed with Chi square or Fisher’s exact test. A *p*-value of < 0.05 was considered significant. All data were analyzed using JMP 15 (Cary, NC, USA). Since this was a retrospective analysis on existing data, a power calculation was not performed to determine the sample size. The primary aim was to compare steroid use (initiated by the providing team) across the three MDF scores based on three control PT values (12, 13.5, and 14.8 s). The secondary aims were to compare each MDF to the MELD in order to predict 30-day survival. Additionally, receiver operating characteristics (ROC) curves for 30-day survival were also calculated among the 3 MDF groups and compared to MELD and FIB-4 while controlling for steroid use.

## 3. Results

**Figure 1:**
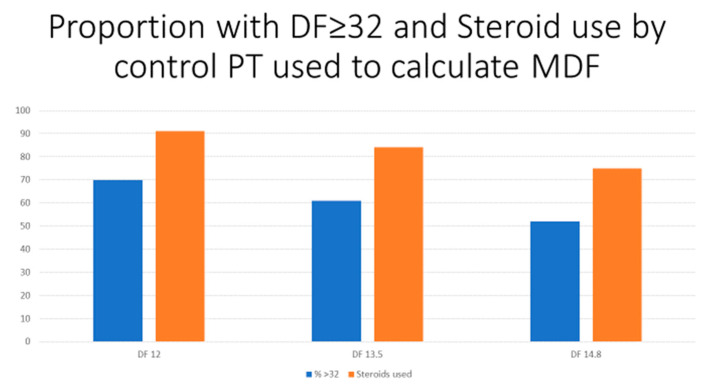
Proportion of MDF ≥ 32 and steroid use for each control PT value via histogram plot. As seen, increases in control PT values resulted in a lower proportion of DF > 32 and a lower proportion of steroid use.

The demographic data in this cohort show a mean age of 48 years, with 60% of the patients being male and 61% of the patients being Caucasian (Table 1). The pertinent laboratory parameters showed a median total bilirubin of 8.75 mg/dL, a mean PT of 21.2 s, and a median creatinine of 0.96 mg/dL. The median MELD of this patient cohort showed a value of 19. The clinical features showed that 12.6% of the patients had HE and 3% had ascites. The median length of hospitalization was seven days, while the median days to death was nine days, in which 138 of the 882 patients would eventually die during the hospitalization. For each patient meeting the ICD-9 and ICD-10 codes of AH, the diagnosis and the decision to treat was under the discretion of the treating physician. For each numerical value of control PT that was used (12, 13.5, and 14.8 s), the percentage of MDF ≥ 32 overall decreased, with increasing control PT values (70%, 61%, and 52%, respectively) (Figure 1, blue bars).

Table 2 and Figure 1 (orange bars) compare steroid use vs. no steroid use among all of the patients who were diagnosed with acute AH and by the control PT that was used in the MDF calculation. Those who received steroids were found to be hospitalized for approximately twelve days compared to those without steroids for eight days, which was statistically significant (*p* < 0.0001). While the 30-day mortality was lower in the steroid group at 14.5% compared to 16.5% in those who did not receive steroids, this was not statistically significant (*p* = 0.41). Those who received steroids were younger (46 vs. 50 years; *p* < 0.0001), female (46% vs. 39%; *p* = 0.05), Caucasian (70% vs. 56%; *p* < 0.0001), and had higher TB (18 mg/dL vs. 7 mg/dL; *p* < 0.0001), INR (2 vs. 1.8; *p* = 0.0001), and MDF ≥ 32 at all of the control PT values (*p* < 0.0001), MELD (24 vs. 18; *p* < 0.0001), and HE (16% vs. 10%; *p* = 0.01).

Table 3 compares those who died to those who survived for at least 30 days. When it was stratified by control PT (12, 13.5, and 14.8 s), the percentage of MDF ≥ 32 was 93%, 87%, 83%, respectively. The mean MELD score was 30 in those who died (*p* < 0.001). Those who died were found to have higher FIB-4 scores (mean 22.3 vs. 14.1, *p* = 0.0052). Higher creatinine levels were also found in the deceased cohort, which was statistically significant (*p* < 0.0001). Those who died were found to have fewer days of hospitalization (mean 9.9 days vs. 10.4 days); however, this was not found to be statistically significant (*p* = 0.69).

When comparing survival at 30 days, we found that the ability of MDF to predict the survival rate was similar across all three of the control PT values that were used to calculate the MDF (AUROC 0.735 for all three control PT values). While the MELD had a higher AUROC (0.767) compared to the MDF, the MDF had a higher AUROC than FIB-4 (0.585).

## 4. Discussion

The MDF is widely used to assess the need for steroids in AH. To our knowledge, this is the first study that investigates whether the control PT truly matters in determining the use of steroids and the overall survival in AH. Our focus in this study was two-fold. We wanted to show that different control PTs will change the percentage of MDF ≥ 32 and, thus, who receive steroids. We also wanted to determine if the control PT that is used in the MDF has an effect on survival. In our study, we observed a similar 30-day survival among each MDF group (by AUROC) and found that steroid use did not impact survival, which corroborates the findings in the former literature on use of steroids in AH [7].

As the numerical value of the control PT increased, the calculated MDF decreased. Expectedly, we found that higher control PT values were used and, thus, lower MDF scores resulted in less steroids being given (Figure 1). This is an important consideration for the providers to keep in mind when they decide which control PT to use in the MDF calculations, as steroids are a class of medications with many known side effects, such as immunosuppression and increased risk of bleed, among others. Acute infection is a hindrance to the use of steroids in those with severe AH [8]. A risk–benefit discussion is often had for patients with acute infection who are indicated for steroid treatment. In these unique circumstances, the addition of N-acetylcysteine to prednisolone therapy in AH has been shown to decrease the rate of infection [9]. More importantly, however, we have found that, while the choice of the control PT that is used can affect treatment decisions, the PT control that is used should not impact the overall survival when using MDF calculations.

Although this study is novel, it supports the recent observations that the MDF is inferior to the MELD in predicting mortality at 30 days no matter what control PT is used [6]. One plausible explanation for its superiority to the MDF is that the MELD considers the creatinine level of the patients. This is important as kidney function is an independent predictor of poorer outcomes in AH, such as mortality [10]. This was found in our study to be statistically significant, as shown in Table 3, with higher creatinine levels in the deceased group of patients who had AH on admission.

On the other hand, there is also new evidence that shows how the MDF, or at least its parameters, have robust utility in the future management of AH. It is well known in the literature that the MELD and the MDF are static scores, meaning that they consider data only at one point in time. In the case of our data, the MDF was calculated on the index admission. However, evidence shows that it is a combination of both dynamic and static scores that is superior to static scores alone [11]. One example of a dynamic score is that of the Lille score, which risk stratifies AH patients who are receiving steroids at seven days into responders vs. non-responders [12]. In order to calculate this score, the age, the creatinine, the PT, the albumin, the initial bilirubin, and the bilirubin at seven days are used, with a value < 0.45 indicating a good response to the steroids in AH patients. This score clearly takes into consideration both parameters from the MELD and the MDF. The Lille score has been found to be the best predictor of mortality for severe disease at seven days in prior studies [13]. Future studies will need to corroborate these findings.

It should be acknowledged that there were several limitations in this retrospective study. Although we believe that we had an adequate sample size, we excluded the patients with missing laboratory parameters on admission, which may have skewed the results. In addition, the decision to diagnose a patient with AH was based on the discretion of the treating physician and not solely on the NIAAA criteria. It is possible that some of the patients who were diagnosed with AH had other causes of acute hepatitis, including autoimmune, infection, and drug-induced liver injury. Some of the patients may have been given the diagnosis of AH without meeting all of the specific exclusion criteria that are defined by NIAAA. While our center does not use steroids if there is an active infection or GI bleeding, initiating steroids was at the discretion of the treating provider and some with those disorders may have been included, therefore affecting our results. Because this was a retrospective analysis, we do not know which control PT was used by the treating team in order to calculate the MDF. Therefore, we did not compare the statistical significance of the different control PT values with each other. While we see a trend towards decreased steroid use with higher control PT values, we are unable to say if this is statistically significant, which may bias our results.

In regard to the other causes of liver enzyme elevation, we found that those with a history of hepatitis C virus (HCV) were less likely to receive steroids than those who did not (5.5% vs. 17.8%, respectively). However, we did not assess for HCV ribonucleic acid (RNA) or the treatment history in these patients, which may also limit our results. Because the percentage of those with a history of HBV was low (1%), we do not think that those with an active (vs. resolved) infection impacted our results. Furthermore, since we did not specifically determine if the subjects had cirrhosis in the setting of AH, we did not differentiate acute on chronic liver failure from AH.

We used clinical criteria to guide our decision on whether the patients truly had AH upon admission or not, and the treatment was ultimately at the discretion of the treating hepatologist. Liver histology is the gold standard for AH. However, because most of the patients did not undergo a liver biopsy, we did not have a histologic confirmation of AH or cirrhosis. On the other hand, there are other studies that have shown that the diagnosis of AH in the setting of high and recent alcohol consumption can confirm the diagnosis in 80% of cases and, therefore, a biopsy is not required [14]. Finally, we did not have 7-day Lille scores in order to assess its clinical impact on steroid use.

In conclusion, to our knowledge, this study is the first of its kind to investigate whether the control PT that is chosen to calculate the MDF has an impact on steroid use and survival. Our findings suggest that, while the choice of the control PT that was used did impact the treatment decisions and the use of steroids, the use of steroids and the choice of the PT control that was used did not impact the overall survival. Similar to recent data, our results show that the MELD outperforms the MDF in terms of predicting short-term survival regardless of the control PT value that is used. However, the MDF still has utility, and the important laboratory parameters that it considers are also used in the Lille score, which is an important prognostic marker in this patient population.

## Figures and Tables

**Table 1:** Demographic and clinical data, *n* = 882 subjects.

Variable	Mean (SD), Median (IQR), or Proportion
Age (years)	48.5 (11.2)
Male/Female (%)	60/40
Race (% White/African American/Asian/Other)	61/29/1/4
Ethnicity (% Non-Hispanic)	92
Weight (kg)	84.7 (25)
Alanine Aminotransferase (ALT) in U/L	57 (32–99.7)
Total bilirubin (mg/dL)	8.75 (3.4–19.75)
Albumin (g/dL)	2.82 (0.67)
Prothrombin time (PT) in seconds	21.2 (7.57)
International Normalized Ratio (INR)	1.86 (0.89)
Creatinine (mg/dL)	0.96 (0.66–1.59)
Platelet count (×1000)	131 (81–198)
Discriminant Function (using PT of 12)	46.7 (28.5–74.0)
Discriminant Function ≥ 32 (%)	70
Discriminant Function (using PT of 13.5)	39.82 (21.6–67.1)
Discriminant Function ≥ 32 (%)	61
Discriminant Function (using PT of 14.8)	33.8 (15.6–61.1)
Discriminant Function > 32 (%)	52
Model for End-Stage Liver Disease (MELD)	19 (12.8–28.0)
FIB-4	8.75 (4.7–15.9)
Hepatic Encephalopathy (%)	12.6
Diabetes Mellitus (%)	18.6
Hepatitis B Virus (%)	1
Hepatitis C Virus (%)	11
Ascites (%)	3
Gastrointestinal bleed (%)	3
Bacteremia (%)	3
Steroids used (%)	45 (*n* = 399)
Died (%)	15 (*n* = 138)
Hospitalization days	7 (3–13)
Days to death (*n* = 138)	9 (4–18.5)

SD = standard deviation, IQR = interquartile range.

**Table 2:** Comparing steroid use.

	Steroid Use	No Steroid Use	*p* Value
Number of patients (*n*)	399	483	
Age (years)	46.2 (11.03) *	50.4 (10.9)	<0.0001
Male/Female (%)	54/46	61/39	0.05
Race (% White/African American/Other)	70/18/12	56/35/9	<0.0001
Ethnicity (% Non-Hispanic)	92	91	0.40
Weight (kg)	85.7 (26)	81.6 (23)	0.0044
Aspartate Aminotransferase (AST) in U/L	353 (1220)	561 (3408)	0.20
Alanine Aminotransferase (ALT) in U/L	122 (449)	148 (480)	0.12
Total bilirubin (mg/dL)	18.5 (11.6)	7.65 (8.3)	<0.0001
Albumin (g/dL)	2.66 (0.54)	2.90 (0.70)	<0.0001
Prothrombin time (seconds)	22.6 (9.1)	20.4 (8.3)	<0.0001
International Normalized Ratio (INR)	2.01 (0.69)	1.78 (1.0)	0.0001
Creatinine (mg/dL)	1.46 (1.36)	1.35 (1.35)	0.22
Discriminant Function (using PT of 12)	67.4 (31)	46.2 (41)	<0.0001
Discriminant Function ≥ 32 (%)	91	53	<0.0001
Discriminant Function (using PT of 13.5)	60 (31)	39 (41)	<0.0001
Discriminant Function ≥ 32 (%)	84	43	<0.0001
Discriminant Function (using PT of 14.8)	54 (30)	34 (39)	<0.0001
Discriminant Function ≥ 32 (%)	75	34	<0.0001
Model for End-Stage Liver Disease (MELD)	24 (9.5)	18 (11.2)	<0.0001
Hepatic Encephalopathy (%)	15.7	10.3	0.01
Diabetes Mellitus (%)	16	20	0.14
Hepatitis B Virus (%)	0.5	1.7	0.11
Hepatitis C Virus (%)	5.5	17.8	<0.0001
Ascites (%)	2	4	0.02
Gastrointestinal bleed (%)	2.6	4.5	0.06
Bacteremia (%)	1.5	4.3	0.015
Died (%)	14.5	16.5	0.41
Hospitalization days	12.56 (13.1)	8.4 (10.4)	<0.0001

* Mean (SD).

**Table 3:** Comparison by 30-day survival.

	Died (30-Day Mortality)	Survived	*p* Value
Number of patients (*n*)	138	744	
Age (years)	49.7 (11.4) *	48.3 (11.1)	0.18
Male/Female (%)	57/43	58/42	0.85
Race (% White/African American/Other)	53/27/20	64/27/9	<0.0001
Ethnicity (% Non-Hispanic)	79	94	<0.0001
Weight (kg)	91 (34)	83 (23)	0.001
Aspartate Aminotransferase (AST) in U/L	1014 (6098)	366 (1195)	0.73
Alanine Aminotransferase (ALT) in U/L	133 (297)	137 (491)	0.37
Total bilirubin (mg/dL)	17.5 (11)	11.6 (11)	<0.0001
Albumin (g/dL)	2.51 (0.58)	2.84 (0.64)	<0.0001
Prothrombin time (seconds)	25.9 (10)	20.6 (6.5)	<0.0001
International Normalized Ratio (INR)	2.41 (1.25)	1.78 (0.76)	<0.0001
Creatinine (mg/dL)	2.16 (1.8)	1.26 (1.2)	<0.0001
Discriminant Function (using PT of 12)	81.6 (48)	51.1 (34)	<0.0001
Discriminant Function ≥ 32	93	66	<0.0001
Discriminant Function (using PT of 13.5)	74.8 (48)	44.2 (34)	<0.0001
Discriminant Function ≥ 32	87	57	<.0001
Discriminant Function (using PT of 14.8)	68.9 (48)	38.2 (34)	<0.0001
Discriminant Function ≥ 32	83	47	<0.0001
Model for End-Stage Liver Disease (MELD)	30 (10.6)	19 (10.2)	<0.0001
FIB-4	22.3 (2.6)	14.1 (1.1)	0.0052
Hepatic Encephalopathy (%)	23	11	<0.0001
Diabetes Mellitus (%)	18	18	0.99
Hepatitis B Virus (%)	0.7	1.2	0.62
Hepatitis C Virus (%)	13	12	0.75
Ascites (%)	2.2	3.5	0.42
Gastrointestinal bleed (%)	5.8	3.1	0.11
Bacteremia (%)	1.4	3.4	0.23
Steroid use (%)	42	46	0.41
Days hospitalized	9.9 (7.6)	10.4 (12.5)	0.69

* Mean (SD).

## Data Availability

No new data were created or analyzed in this study. Data sharing is not applicable to this article.
